# The impact of language discordance on genetic counselors' ability to establish a working alliance with patients

**DOI:** 10.1002/jgc4.70019

**Published:** 2025-04-30

**Authors:** Anna Burton, Dana Schlegel, Charité Ricker, Beverly M. Yashar

**Affiliations:** ^1^ Department of Human Genetics University of Michigan Ann Arbor Michigan USA; ^2^ Kellogg Eye Center Michigan Medicine Ann Arbor Michigan USA; ^3^ Norris Comprehensive Cancer Center University of Southern California Los Angeles California USA

**Keywords:** client centered communication, contracting success, genetic counselor ‐ interpreter relationship, health disparities, language discordance

## Abstract

We explored the impact of language discordance (LD) on quality of care by asking genetic counselors (GCs) about their perception of how their lack of proficiency in a patient's language affects their sessions. We hypothesized that contracting, which relies on ongoing, bidirectional communication between GC and patient, is particularly vulnerable to LD. Specifically, we evaluated the impact of dialogue engagement (whether GCs ranked dialogue as more one‐sided/rigid or more interactive/conversational), time sufficiency (how adequate the GCs ranked the time allotted for the session), and interpreter‐related factors (experience and relationship with interpreters; perceived ability and knowledge of how to work with interpreters) on GCs' perceived ability to contract in LD sessions. Forty‐five GCs recruited from the NSGC listserv completed a 42‐item survey exploring these topics through reflection on their most recent LD and language concordant (LC) sessions. The outcome measure of “perceived contracting success” was defined based on five practice‐based competencies. Results were analyzed using Wilcoxon signed ranks tests and linear regressions and found that GCs' perceived (1) contracting success, (2) dialogue engagement, and (3) time sufficiency were significantly lower in LD sessions (*p* < 0.001 for all 3). Perceived contracting success in LD sessions had a positive relationship with both perceived dialogue engagement and perceived time sufficiency (*r* ^2^ = 0.312, 0.103). Also, perceived dialogue engagement increased with higher perceived time sufficiency and trust in the interpreter (*r* ^2^ = 0.235, 0.27). Our study is the first to quantitatively explore factors impacting perceived contracting success in LD GC sessions and suggests that LD may hinder communication and session tailoring. This highlights the importance of GCs being more intentional about having interactive dialogue with patients in LD sessions, considering allotting more time for LD sessions, and meeting with the interpreter prior to LD sessions to establish a trusting relationship.

## 1 INTRODUCTION

### 1.1 Background and rationale

There is a large discrepancy between the diversity of the patient population in the United States and the demographic makeup of the genetic counselors serving them. While 90% of genetic counselors identify as non‐Hispanic White, almost half of the individuals in the United States identify as a race/ethnicity *other than* non‐Hispanic White (NSGC PSS, 2021; US Census Bureau, 2015). Similarly, only 14% of genetic counselors are fluent in a language other than English, yet 25 million people in the United States speak English less than “very well” (NSGC PSS, 2021; US Census Bureau, 2015). Given the discrepancies highlighted by these statistics, language discordance (LD) often emerges. LD describes situations in which the genetic counselor and patient are not proficient in the same language, thus requiring some form of interpretation. An important question then arises: What are the impacts of LD on the quality of care delivered to patients with limited English proficiency (LEP)?

Research of both genetic counseling and multiple other specialties shows that LD can negatively influence patients' experiences in a healthcare setting and make them feel vulnerable, disempowered, and frustrated (Luksic et al., 2020; Yeheskel & Rawal, 2019). Specifically, LD is associated with patients having difficulty understanding their medical condition, expressing their concerns, asking questions, participating in the decision‐making process, and establishing relationships with their providers (Al Shamsi et al., 2020; Jaramillo et al., 2016; Kamara et al., 2018; Yeheskel & Rawal, 2019). A recent meta‐analysis suggests that LD is also associated with poorer clinical health outcomes, including decreased uptake of preventative cancer screening, poorer control of diabetes, and lower rates of adequate psychiatric care (Cano‐Ibáñez et al., 2021).

Medical interpreters have an invaluable skillset that can help mediate the impacts of LD and facilitate communication between genetic counselors and their patients. They are integral members of the healthcare team and take on roles as patient advocates, cultural brokers, and emotional supporters. However, research suggests that there are inconsistencies in genetic counselors' and interpreters' perspectives on the role of interpreters during sessions, as well as a lack of trust between genetic counselors and interpreters (Lara‐Otero et al., 2018; Wang et al., 2022). Many providers also feel that consultation duration is significantly increased when interpreters are used, which raises the concern that time constraints may be posing an additional barrier to the quality of care being delivered to patients with LEP (Jaeger et al., 2019; White et al., 2018).

While there are several distinct components of a genetic counseling session that could be negatively impacted by language discordance, there is one that stands out as being particularly vulnerable in this context and therefore pivotal to explore: contracting. Contracting is “the two‐way communication process between the genetic counselor and the patient/client which aims to clarify both parties' expectations and goals for the session” (Doyle et al., 2016). Contracting is the first step in forming a working alliance and positive relationship between the genetic counselor and the patient. This relationship is central to the genetic counseling process, as it enables patients to feel “supported, cared about, connected, and validated,” according to the Reciprocal‐Engagement Model (REM), which is the framework that defines core principles of genetic counseling practice (Redlinger‐Grosse et al., 2017; Veach et al., 2007). Contracting is also a key component of tailoring a session to each individual patient's unique needs and concerns. This personalization is essential to delivering the client‐centered care that is so valued by the genetic counseling profession (Doyle et al., 2016; Redlinger‐Grosse et al., 2017; Veach et al., 2007). Successful contracting requires the genetic counselor to elicit and incorporate each client's specific expectations, perceptions, knowledge, and concerns during the session, which relies upon back‐and‐forth dialogue with the patient (Doyle et al., 2016). Dynamic communication with patients to elicit this information can be time consuming and challenging for all patients and would be expected to be even more difficult when the additional challenge of LD is present in the session. Thus, it was hypothesized that contracting is particularly susceptible to LD.

### 1.2 Objectives

The goal of this study was to define critical parameters impacting genetic counselors' ability to establish a tailored, patient‐centered working alliance with patients when professional medical interpreters are used. By identifying and exploring the factors that influence genetic counselors' ability to contract with patients in LD sessions, we hoped to pinpoint ways through which genetic counseling services can be more equitable for patients, regardless of the language they use. This will become increasingly important given that the linguistic diversity of the patient population in the United States is already far greater than that of genetic counselors, and it is expected to rise significantly more in the coming decades (Hallford et al., 2020; U.S. Census Bureau, 2015; Vespa et al., 2018).

## 2 METHODS

### 2.1 Institutional review board approval and informed consent

This study was reviewed by the Health Sciences and Behavioral Sciences Institutional Review Board (IRB‐HSBS) at the University of Michigan and was granted “Not regulated” status on 4/08/2021 (HUM00197180). In keeping with the IRB approved not regulated status of this human subjects research, a waiver of documentation of informed consent was obtained from all research participants at the time of study participation.

### 2.2 Participants

Board‐certified genetic counselors who conducted at least one language discordant (LD) and one language concordant (LC) direct patient care session within the past 12 months were eligible to participate in this survey‐based study. LD sessions were defined as those during which a professional medical interpreter (either in‐person, over the phone, or via video) was used. LC sessions were defined as those during which the genetic counselor and patient communicated using the same language, not requiring a professional medical interpreter or any other person or device to assist with interpretation/translation during the visit. We defined professional medical interpreters as individuals who are trained in medical interpretation and appointed or contracted by a hospital, medical center, or health care clinic to assist in communicating with patients who have limited proficiency in the language spoken by the provider, or who are Deaf or hard of hearing. Therefore, sessions involving the use of a professional medical interpreter of American Sign Language (ASL) or any spoken language were considered to be LD sessions. Family members or friends of a patient who assisted with interpretation during a session were not considered professional medical interpreters, and sessions interpreted by these individuals were not included in our study.

### 2.3 Setting and recruitment

Participants were recruited from the National Society of Genetic Counselors (NSGC) and the Michigan Association of Genetic Counselors (MAGC). An email announcement was sent to the NSGC listserv on November 10, 2021 and December 1, 2021 and to the MAGC listserv on November 17, 2021. These emails contained a short description of the study as well as a Qualtrics weblink for survey completion. Data collection took place from November 10, 2021, until December 9, 2021. After survey completion, participants were entered into a raffle to win twenty‐five $20 monetary rewards.

### 2.4 Study design

A 42‐item survey was developed and composed of five main sections: (1) eligibility for participation, (2) demographics, (3) beliefs regarding how providers should work with interpreters, and (4 and 5) reflection on the GC's most recent LD and LC session. Questions were designed to elicit responses evaluating participants' perceived ability to contract during each of those encounters, as well as various factors that we hypothesized might impact their perceived contracting ability. Many questions utilized the semantic differential scale developed by CE Osgood, which involves asking participants to rate factors on a gradient scale with two opposing adjectives at each end (Osgood, 1952, 1962). However, our survey instrument was novel and was not derived from previously validated tools.

Given that contracting during a session does not always occur between the genetic counselor and patient (i.e., in the case of a young pediatric patient or individual with intellectual disability), the term “primary communicator” was used throughout the survey, which was defined as the person with whom the genetic counselor interacted the most throughout the session, whether that be the patient or someone accompanying the patient. The survey was designed with input from a professional American Sign Language interpreter and three genetic counselors, one who previously worked as a professional medical interpreter and one who regularly provides LC patient care in Spanish. A pilot survey was reviewed by two genetic counselors and one genetic counseling student and revised according to their feedback.

### 2.5 Variables and hypotheses

**FIGURE 1:**
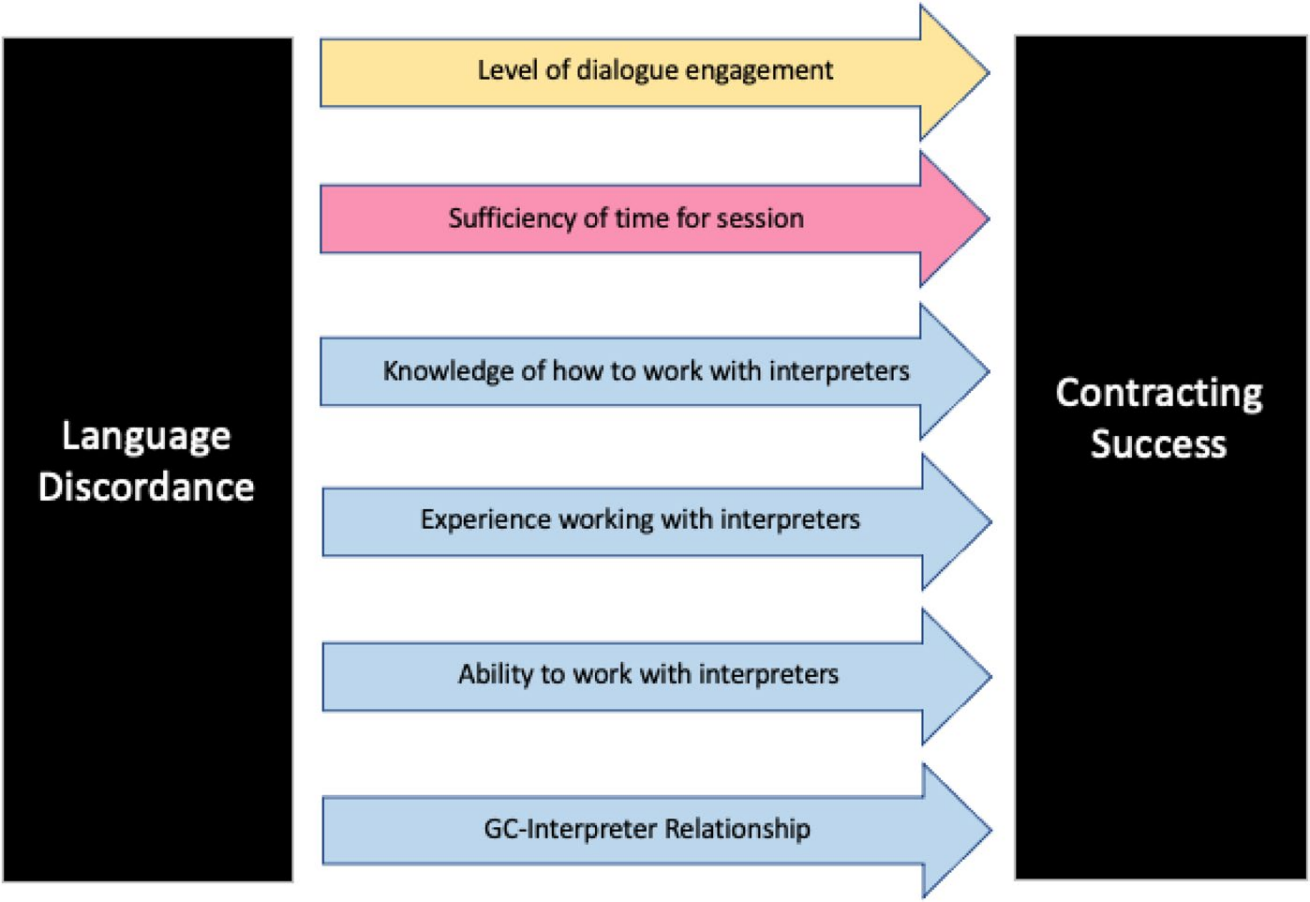
Research Model. The three categories of factors hypothesized to impede contracting success in LD sessions (yellow: Perceived level of dialogue engagement during the session; pink: Perceived sufficiency of time allotted for session; blue: Various interpreter‐related factors).

Specifically, this study sought to (1) distinguish differences in perceived contracting success, dialogue engagement, and time sufficiency between LD and LC sessions and (2) evaluate the impact(s) of perceived dialogue engagement, perceived time constraints, and various interpreter‐related factors on genetic counselors' perceived ability to contract in LD versus LC sessions (Figure 1). Genetic counselors' perceived ability to contract was the dependent variable in this study while perceived dialogue engagement, perceived time constraints, and various interpreter‐related factors were the variables we evaluated as predictors of the dependent variable.

These research questions resulted in the development of four hypotheses. First, we hypothesized that LD would be associated with lower perceived contracting success based on previous literature suggesting patients with limited English proficiency have a difficult time expressing their concerns and establishing relationships with their providers (Al Shamsi et al., 2020; Jaramillo et al., 2016; Kamara et al., 2018; Yeheskel & Rawal, 2019). Second, we hypothesized that LD would be associated with lower levels of dialogue engagement and would impact contracting success based on previous studies that demonstrated there is less interactive, relationship‐enhancing communication and fewer questions asked by patients in LD sessions (Ault et al., 2019; de Leon et al., 2022; Dunlap et al., 2015; Jaramillo et al., 2016). Third, we hypothesized that LD would be associated with lower perceived sufficiency of time allotted for the session and would impact contracting success based on previous studies indicating that many clinicians believe that the use of an interpreter significantly increases consultation duration (Jaeger et al., 2019; White et al., 2018). Lastly, we hypothesized that various interpreter‐related factors would impact perceived contracting success in LD sessions based on previous studies highlighting genetic counselors' lack of trust in interpreters, as well as lack of understanding of their role in the session (Lara‐Otero et al., 2018; Wang et al., 2022). These hypotheses were used to generate our study model (Figure 1).

### 2.6 Data sources/ measurement

To explore participants' perceived ability to contract, we evaluated five separate components of this task, which were based on the practice‐based competencies for genetic counselors: (i) the ability to elicit patient expectations and perceptions, (ii) the ability to elicit patient knowledge, (iii) the ability to elicit patient concerns, (iv) the ability to incorporate these components into a mutually agreed upon agenda, and (v) the ability to modify the agenda throughout the session (Doyle et al., 2016). Perceived contracting success was chosen as the outcome measure, given that it is one of the first steps of forming a patient‐centered alliance and building a supportive patient‐provider relationship, which is a central tenet of the Reciprocal‐Engagement Model (REM) of genetic counseling practice (Redlinger‐Grosse et al., 2017; Veach et al., 2007). Additionally, high‐quality contracting allows genetic counselors to tailor sessions to patients' individual, unique needs. Perceived success at achieving each component of contracting was based on participants' Likert responses to five separate statements related to each of the five elements of contracting. Overall contracting success scores for LD and LC sessions were calculated by summing the scores of each of the five components of contracting described above. Each of these components is essential to contract with patients, thus supporting our decision to evaluate contracting as an aggregate entity as opposed to assessing each of its components individually.

Other survey questions were designed to explore various factors that may impact genetic counselors' perceived ability to achieve the five contracting‐related competencies described above. The first factor evaluated was genetic counselors' knowledge of how to work with interpreters. This was measured by the accuracy of their answers to three questions about how the International Medical Interpreters Association (IMIA, n.d.) recommends healthcare providers engage with interpreters. Other factors included genetic counselors' experience working with interpreters, perceived ability to work with interpreters, and perceived relationship with the interpreter used in their most recent LD session. The survey also contained questions evaluating genetic counselors' perceived level of dialogue engagement with the primary communicator during their most recent LC and LD sessions, as well as the perceived sufficiency of time allotted for each of these sessions. Most of these factors were measured using the semantic differential scale developed by CE Osgood, which involves asking participants to rate each factor on a gradient scale with two opposing adjectives at each end (Osgood, 1952, 1962). Specifically, to quantify dialogue engagement, participants were asked to rank the dialogue between themselves and the primary communicator during the session on a scale between two opposing sets of adjectives (“one‐sided” vs. “interactive” and “rigid” vs. “conversational”). Similarly, time sufficiency was quantified by participants ranking their perception of the amount of time allotted for the session on a scale between “not sufficient” and “sufficient.”

### 2.7 Bias

In order to address the potential for recall bias, inclusion criteria for survey participation stipulated that the most recent LD and LC sessions must have occurred within 12 months of survey completion. This date range was chosen to maximize both participant eligibility and recall, given that some genetic counselors may not frequently work with patients with limited English proficiency.

To address sampling bias, the survey was made accessible to all members of the National Society of Genetic Counselors by recruiting participants through that organization's listserv. Lastly, design bias was addressed by creating the survey with input from a professional American Sign Language interpreter and three genetic counselors, one who previously worked as a professional medical interpreter and one who regularly provides LC patient care in Spanish. A pilot survey was also reviewed by two genetic counselors and one genetic counseling student and revised according to their feedback.

### 2.8 Study size

This study was open to all genetic counselors who had provided both LD and LC genetic counseling in the 12‐month period prior to the window of survey recruitment from November 10, 2021 to December 1, 2021.

### 2.9 Quantitative variables and statistical methods

Quantitative statistical analysis was completed with the help of consultants at the University of Michigan Center for Statistical Consultation and Research (CSCAR) using SPSS statistical software version 28. For all analyses, 0.05 was used as the threshold to discriminate significant from non‐significant results.

Gender and race/ethnicity demographics of the study population were compared to those of the National Society of Genetic Counselors (NSGC) population, based on the 2021 Professional Status Survey (PSS), using a Fisher's Exact Test. This test determined if the racial and ethnic diversity of our study population was representative of the larger NSGC community and was selected specifically given the small sample size and lack of normal distribution of our data. These data were collected with more granularity in terms of self‐identified race/ethnicity (Figure S1), but for statistical purposes, were collapsed into two categories: non‐Hispanic White and all others (Figure S5).

Each of the five components of the outcome measure (perceived contracting success) was scored and given a numerical value based on participant responses to the Likert‐style questions asked (1, 2, 3, 4, or 5). The scores for each of these five components were summed to yield an overall “success” score of establishing a mutually agreed upon agenda (possible range of 5–25). Similarly, scores for genetic counselors' perceived level of dialogue engagement with the primary communicator in their most recent LC and LD sessions, as well as for the perceived sufficiency of time allotted for each of these sessions, were calculated based on their responses to the semantic differential questions. For example, for the allotted time variable, participants were prompted to select an integer between −5 at one end of the scale (indicating insufficient) and +5 at the other end (indicating sufficient). To determine if perceived contracting success, perceived level of dialogue engagement with the primary communicator, and perceived sufficiency of time for the session differed between LC and LD sessions, Wilcoxon signed ranks test with two related samples were performed. This test was selected rather than a *t*‐test given the small sample size and the fact that participant responses were not normally distributed.

To determine if overall contracting success (summed score 5–25 based on Likert responses) was predicted by any of the factors hypothesized to have an impact on this outcome measure, a series of linear regressions were performed. Linear regressions were used given that we wanted to determine the average change in contracting success (the dependent variable) based on one‐unit changes in each of the independent variables. However, for the question about respecting the judgment of interpreters during sessions, which had true/false/unsure answer options instead of an integer value, an answer of “true” versus an answer of “false” or “unsure” was compared using an independent‐samples *t*‐test. It was confirmed that these responses had a normal distribution, which is why a *t*‐test was used specifically rather than a non‐parametric test.

## 3 RESULTS

### 3.1 Participants

A total of 46 individuals completed the survey, and 45 responses were analyzed. One participant's responses were not included in the analysis because that they were deemed inconsistent with an accurate understanding of the definition of a LD session. Specifically, English was listed as the language spoken by the patient during the LD session, yet English was also listed as the only language spoken by the genetic counselor.

### 3.2 Descriptive data

All participants identified as women (45/45, 100%) and the majority were non‐Hispanic White (36/45, 80%). Other races/ethnicities reported included Hispanic/Latinx, East, South, and Southeast Asian, and other. There was no significant difference between the distribution of gender and race/ethnicity in the study population and that of the National Society of Genetic Counselors (NSGC) population (Figure S5, *p* = 0.169 and *p* = 0.33, respectively). Participants had a wide range in the years of experience providing direct patient care and worked in a variety of specialties, including prenatal, cancer, pediatrics, and others (Figures S1 and S2).

English was the language used in the majority of the LC sessions (44/45, 97.8%), but one LC session was conducted in Spanish (2.2%). The majority of primary communicators in the LD sessions used Spanish (32/45, 71.1%), but other languages included Albanian, American Sign Language (ASL), Arabic, Farsi, French, Haitian Creole, Japanese, Kinyarwanda, Korean, Polish, Portuguese, Russian, and Tigrinya (1/45, 2.2% for each; Figure S3). Approximately 50% of both LD and LC sessions occurred in‐person, while the other half occurred either over the phone or via video. Most professional medical interpreters used in the LD session were telephone interpreters (28/45, 62.2%), but some sessions utilized an in‐person interpreter (8/45, 17.8%), video interpreter (8/45, 17.8%), or a combination of a telephone and an in‐person interpreter (1/45, 2.2%; Figure S2).

### 3.3 Outcome data and main results

#### 3.3.1 Language discordance is associated with lower perceived contracting success

**FIGURE 2:**
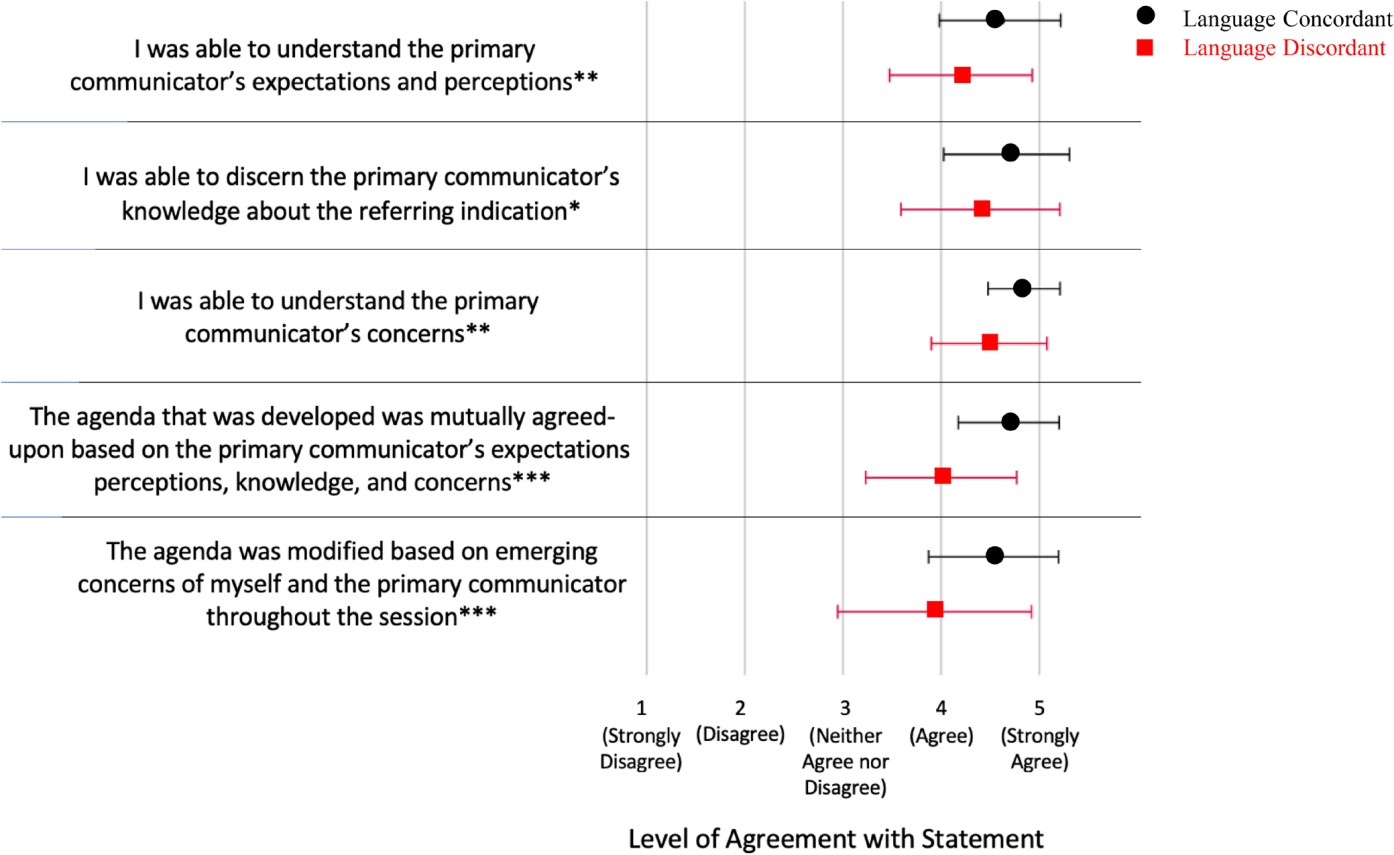
Genetic counselors' perceived contracting success was significantly lower in interpreter‐mediated, LD (LD) sessions than in non‐interpreter‐mediated, LC (LC) sessions in all five elements of contracting. X‐axis shows participants' Likert responses (mean ± SD on a 1–5 scale) to the statements about contracting on the Y‐axis. Asterisks next to statements on left denote statistical significance (one asterisk (*) denotes a *p*‐value <0.05, two asterisks (**) denote a *p*‐value <0.01, and three asterisks (***) denote a *p*‐value <0.001).

To determine the impact of LD on contracting success, we compared perceived success in achieving each of the five individual components of contracting, as well as overall contracting success (a sum of all components) for LD and LC sessions. In all aspects of contracting, genetic counselors' perceived contracting success was significantly lower in interpreter‐mediated, language‐discordant (LD) sessions than in non‐interpreter‐mediated, LC (LC) sessions (Figure 2). These differences were statistically significant in all five elements of contracting (*p* = 0.004, 0.029, 0.002, <0.001, and <0.001, from top to bottom). Overall perceived contracting success (mean ± SD) was significantly lower in LD than in LC sessions as well (21.0 ± 2.5 for LD vs. 23.3 ± 2 for LC; *p* < 0.001).

#### 3.3.2 Language discordance is associated with lower perceived dialogue engagement, which predicted overall contracting success

**FIGURE 3:**
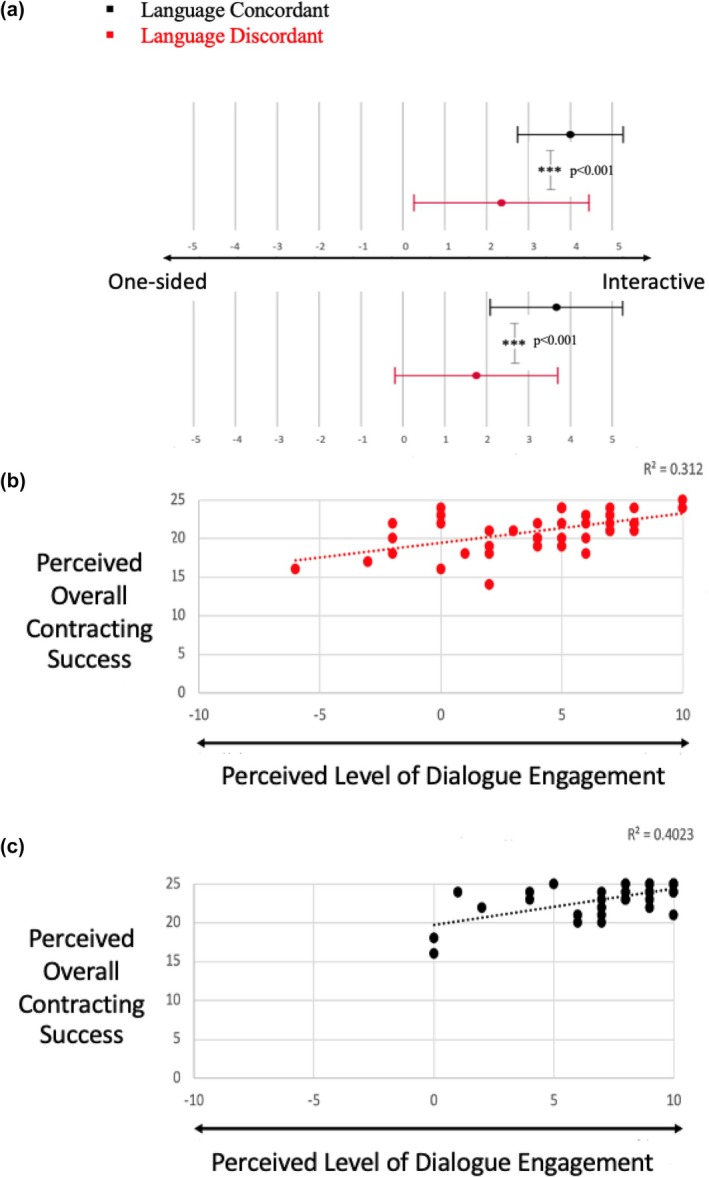
LD is associated with decreased dialogue engagement, which predicts contracting success. (a) Genetic counselors' perceived level of dialogue engagement with the primary communicator was significantly lower in the interpreter‐mediated, LD sessions than in the LC sessions. X‐axis shows participant semantic differential response (mean ± SD) on a −5 to +5 scale, with two opposing adjectives describing dialogue. Asterisks denote statistical significance (three asterisks (***) denote a *p*‐value <0.001). (b) Genetic counselors' perceived level of dialogue engagement with the primary communicator predicted their perceived contracting success in both LD and (c) LC sessions.

To quantify dialogue engagement, our participants were asked to describe the dialogue between themselves and the primary communicator during the session using *two* separate sets of adjectives (one‐sided vs. interactive *and* rigid vs. conversational). Consistent with our hypothesis, genetic counselors perceived the dialogue with the primary communicator as less interactive and less conversational in LD sessions than in LC sessions (Figure 3a, *p* < 0.001 for both the one‐sided vs. interactive scale and the rigid vs. conversational scale).

When examining whether dialogue engagement predicted overall contracting success, we combined the dialogue engagement scores from each of the two scales to yield an overall dialogue engagement score ranging from −10 to +10. The decision to use this aggregate score was made given that responses to both dialogue engagement scales were moderately correlated in the LD sessions (Pearson correlation = 0.648) and strongly correlated in the LC sessions (Pearson correlation = 0.809). In both LD (LD) and LC (LC) sessions, the genetic counselors' perceived level of dialogue engagement was positively correlated with their perceived overall contracting success (Figure 3b,c, LD: *p* < 0.001, *r* ^2^ = 0.312; LC: *p* = 0.026; *r* ^2^ = 0.402). Together, these data led us to conclude that LD is associated with lower perceived level of dialogue engagement, which is a predictor of perceived contracting success.

#### 3.3.3 Language discordance is associated with lower perceived sufficiency of time allotted for session, which predicted overall contracting success

**FIGURE 4:**
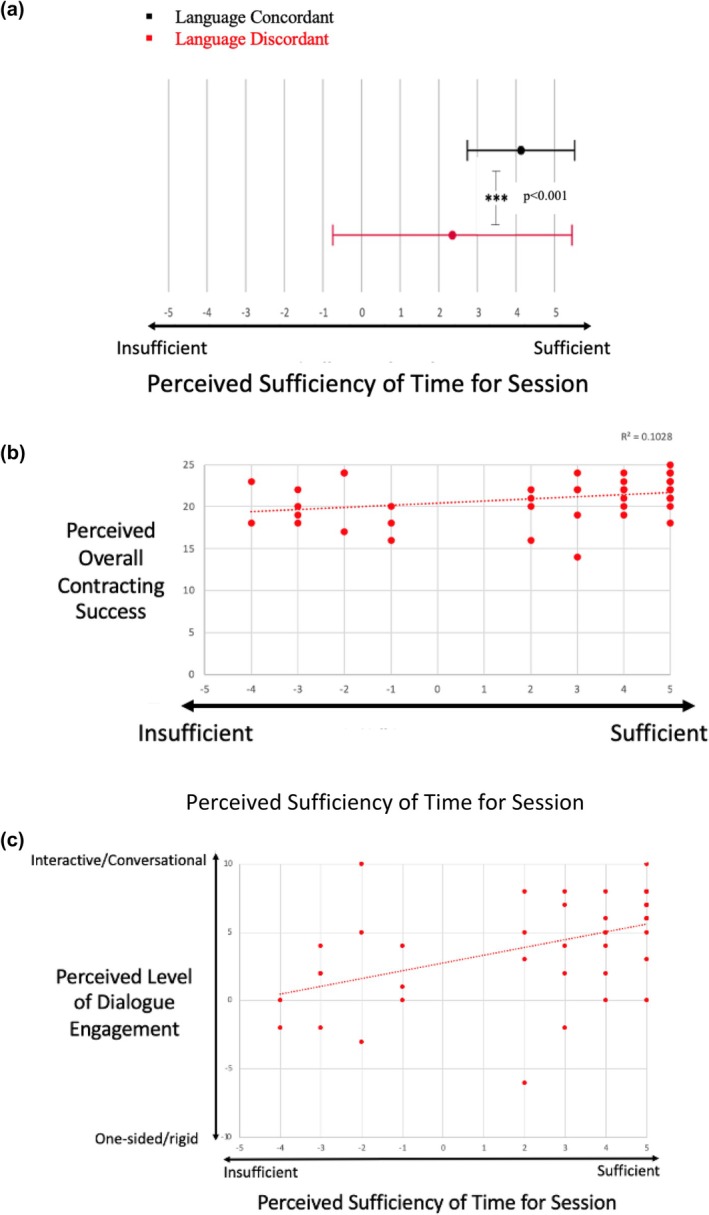
Impact of time constraints in LD sessions. (a) Genetic counselors' perceived sufficiency of time for the session was significantly lower in the interpreter‐mediated, LD sessions than in the LC sessions. X‐axis shows participant semantic differential response (mean ± SD) on a −5 to +5 scale, with two opposing adjectives describing time sufficiency. Asterisks denote statistical significance (three asterisks (***) denote a *p*‐value <0.001). (b) Genetic counselors' perceived sufficiency of time allotted for the session predicted their perceived contracting success and (c) their perceived dialogue engagement in LD sessions.

To test our hypothesis that LD would be associated with lower perceived sufficiency of time allotted for the session, we examined the impact of perceived time constraints on genetic counselors' perceived ability to contract in LD sessions compared to LC sessions. Consistent with our hypothesis, genetic counselors perceived the time allotted for the LD sessions to be less sufficient than the time allotted for the LC sessions (Figure 4a, *p* < 0.001). Additionally, genetic counselors' perceived sufficiency of time allotted for the session was predictive of perceived contracting success in LD sessions (Figure 4b, *p* = 0.032, *r* ^2^ = 0.103). However, time sufficiency was not as strong of a predictor of contracting success, as was dialogue engagement. This is reflected by the lower *r* ^2^ value based on our data, as well as our f‐test results (comparing two nested linear regression models). The *f*‐test indicated that the addition of the dialogue engagement variable significantly improved our model's ability to predict contracting success in LD sessions, while addition of the time sufficiency variable did not significantly improve our model's predictive ability in these sessions (*p* < 0.001 vs. *p* = 0.659). These results overall suggest that perceived dialogue engagement was more important in predicting contracting success than was perceived time sufficiency in LD sessions. In LC sessions, on the other hand, the genetic counselors' perceived sufficiency of time allotted was not at all correlated with their ability to contract (*p* = 0.073, *r* ^2^ = 0.073).

#### 3.3.4 Perceived level of trust with the interpreter predicted perceived dialogue engagement in language‐discordant sessions

**FIGURE 5:**
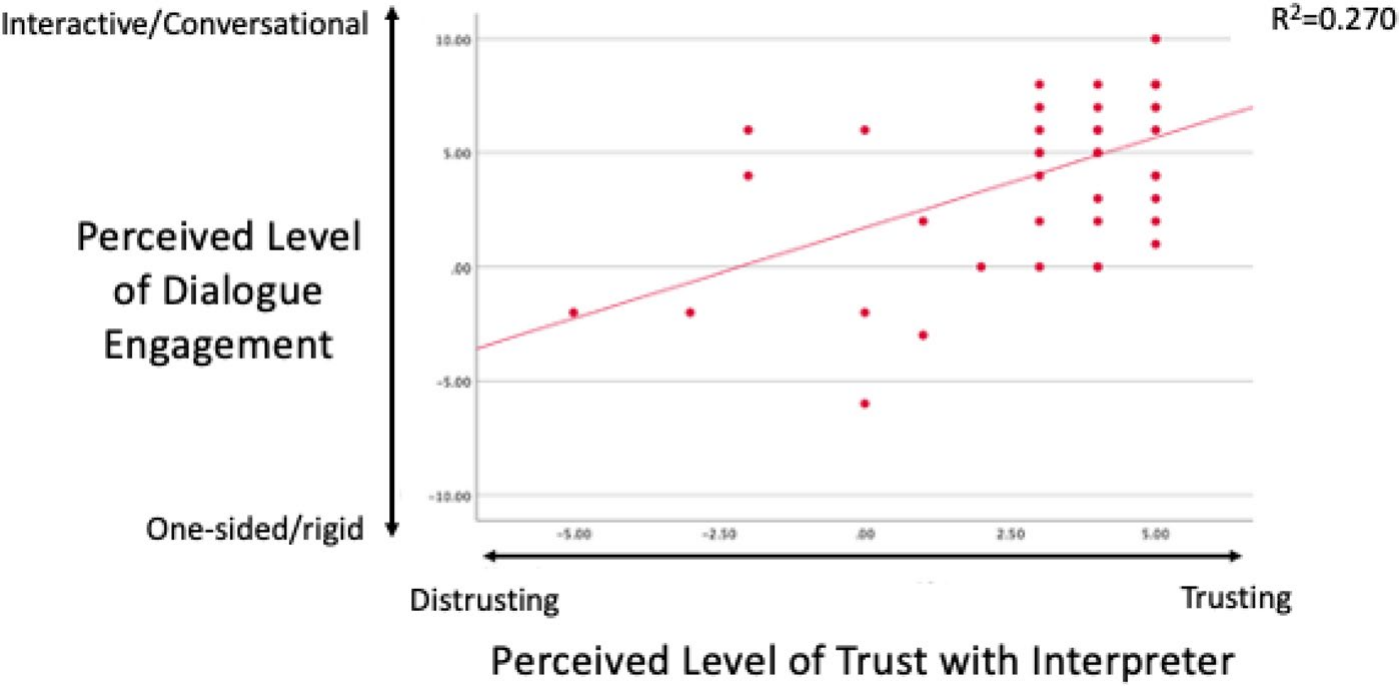
Trust with the interpreter impacts dialogue with the patient. Genetic counselors' perceived level of trust with the interpreter was predictive of their perceived level of dialogue engagement with the primary communicator during LD sessions.

Solely evaluating how dialogue between patient and genetic counselor and logistical factors like time constrains impact contracting success would be incomprehensive, given that a third party, the interpreter, is involved in LD sessions. We therefore evaluated the impact of multiple factors relating to the interpreter on contracting success (our outcome measure) as well as on dialogue engagement (the best predictor of contracting success). These factors included genetic counselors' knowledge of how to work with interpreters, previous experience working with interpreters, perceived ability to work with interpreters, and perceived relationship with interpreters. None of these factors predicted overall contracting success in LD sessions, and the only factor that predicted perceived dialogue engagement in LD sessions was perceived level of trust in the interpreter (Figure 5, *p* < 0.001, *r* ^2^ = 0.270).

Surprisingly, we found that genetic counselors who answered “false” or “unsure” to the statement “During a session, if the interpreter insists that a question is inappropriate, his/ her judgement should be respected” had a significantly higher perceived ability to understand the primary communicators' expectations and perceptions than did genetic counselors who answered “true” (Figure S4). The mean scores for understanding expectations and perceptions were 4.3 in those who answered “false” or “unsure” compared to 3.8 for those who answered “true” (*p* = 0.047).

### 3.4 Other analyses

The results from testing our hypotheses suggested that the best predictor of contracting success is perceived dialogue engagement. Consequently, we felt that it was important to also examine if time sufficiency or any interpreter‐related factors might predict dialogue engagement. We found that genetic counselors' perceived sufficiency of time allotted for the session predicted their perceived level of dialogue engagement in LD sessions (Figure 4c, *p* < 0.001; *r* ^2^ = 0.235). In LC sessions, on the other hand, there was no correlation between perceived time sufficiency and dialogue engagement (*p* = 0.065; *r* ^2^ = 0.077). Together, these results led us to conclude that having sufficient time for a session is perceived as critical when working with patients with LEP, as it predicts both contracting success and dialogue engagement in LD sessions.

## 4 DISCUSSION

### 4.1 Key results

To our knowledge, this is the first study to quantitatively explore the factors that impact genetic counselors' perceived contracting success with their patients specifically in the context of LD. Our results indicated that genetic counselors' perceived contracting success, dialogue engagement, and sufficiency of time for the session were lower in LD than in LC sessions. Our data also indicated that dialogue engagement was the best predictor of perceived contracting success, and that time sufficiency predicted both dialogue engagement and perceived contracting success. Additionally, genetic counselors' perceived relationship with the interpreter (specifically, level of trust and respect for their judgment) appeared to be more important than other interpreter‐related factors in terms of dialogue engagement and success with certain aspects of contracting.

### 4.2 Interpretation

Overall, our results suggest that when there is LD, genetic counselors believe they communicate less effectively and have a harder time eliciting and incorporating patient expectations, perceptions, knowledge, and concerns into the session. This ultimately suggests that LD genetic counseling sessions may be less tailored to each individual patient's unique needs and concerns. Time constraints and a negative relationship with the interpreter also seem to hinder the genetic counselors' ability to effectively communicate with LD patients to understand and best address their needs.

Based on the results of our study, genetic counselors reported dialogue to be less interactive and conversational in interpreter‐mediated, LD sessions than in non‐interpreter‐mediated, LC sessions. This finding was expected, as it is consistent with previous studies. For example, an audio recording‐based study suggested there were diminished levels of interactive communication between genetic counselors and patients when medical interpreters were used. Specifically, LD sessions began with a more fact‐based, information‐gathering tone, and included less “non‐essential dialogue” to elicit patients' stories (Ault et al., 2019). Additionally, a qualitative study exploring Spanish‐speaking genetic counselors' perspectives on LD revealed that genetic counselors perceive the communication to be less accurate, efficient, complex, and relationship‐enhancing in LD sessions (de Leon et al., 2022). Patients who receive LD care also tend to ask fewer questions, which is another indication that they may be less engaged in conversation (Dunlap et al., 2015; Jaramillo et al., 2016). Unsurprisingly, a recent qualitative study suggested that Spanish‐speaking patients' were less afraid to ask questions during LC genetic counseling sessions (Jimenez et al., 2022). The results of our study contribute to the existing literature by further highlighting the association between LD and diminished conversational engagement.

What is most concerning about the decreased interactive communication in LD sessions is that it has the potential to hinder genetic counselors' ability to deliver tailored, patient‐centered care to individuals with LEP. Specifically, our study indicated that perceived dialogue engagement was predictive of perceived contracting success and that both of these outcomes were diminished in LD sessions. It is not surprising that there was a relationship between dialogue engagement and contracting success, given that contracting is an interactive, dynamic process that involves ongoing communication throughout the session. Many studies highlight the importance of taking the time to inquire about patient motivations for seeking genetic counseling and evaluating their baseline knowledge, as gathering this information can shift how the genetic counselor frames the conversation, prioritizes what to discuss, and makes recommendations (Marzulla et al., 2021; Schmidlen et al., 2014; Schmidt et al., 2019). The findings that genetic counselors perceive dialogue as less interactive and conversational and contracting as less successful in interpreter‐mediated sessions suggest that patients in LD sessions may be receiving genetic counseling care that is less tailored and personalized than patients in LC sessions. It is critical that genetic counselors be intentional in having interactive conversations with patients with LEP to better elicit and address their specific needs and concerns, whether they be educational and/or psychosocial. Also, it is important to note that communicating about patient concerns should occur throughout the entire session, not just at the beginning. This point is illustrated by a study of genetic counseling for psychiatric disorders, which indicated that almost 20% of patients changed their mind about what they hoped to discuss during the visit *after* the initial contracting at the beginning of the session (Borle et al., 2018).

Aside from being more intentional about communicating and contracting with patients with LEP, it may also be beneficial to allot more time for sessions that involve an interpreter. Our results suggest that perceived time constraints make it harder for genetic counselors to effectively communicate with patients to understand and address their concerns. Unsurprisingly, genetic counselors felt that the time allotted for LD sessions was less sufficient than that allotted for LC sessions. This too is consistent with previous studies that revealed that many clinicians believe that the use of an interpreter significantly increases consultation duration (Jaeger et al., 2019; White et al., 2018). Interestingly, an audio recording‐based study found no significant difference in session length, yet a decrease in words used during LD than in LC sessions (Ault et al., 2019). It is possible that genetic counselors believe time is less sufficient for interpreter‐mediated sessions, yet are constrained by busy schedules and workflow logistics that prevent them from spending more time with patients with LEP, even if they feel it is needed. Hmong interpreters have also identified time constraints as a contributing factor in creating difficult interpretation experiences (Krieger et al., 2018). It is important to note that, based on the results of our study, if genetic counselors felt the time allotted for a LD session was more sufficient, they also felt that the dialogue was more interactive and conversational and that the contracting was more successful. Thus, time constraints certainly seem to contribute to the communication problems associated with LD. Perhaps allotting more time for LD sessions could be helpful in improving communication and contracting, ultimately enabling genetic counselors to better and more pointedly meet the individual needs of patients with LEP.

When discussing LD, it is imperative to consider the role that interpreters play in the communication process between genetic counselors and patients. Our data suggest that genetic counselors' perceived relationship with the interpreter (specifically level of trust and respect for their judgment) is the most important interpreter‐related factor in terms of genetic counselors' ability to communicate and contract with patients. Previous studies have illustrated that there are discrepancies in genetic counselors' versus interpreters' understanding of the purpose of interpreters during patient encounters, with interpreters viewing their role beyond strictly a conduit of verbatim information (Hadziabdic & Hjelm, 2013; Hsieh, 2008; Lara‐Otero et al., 2018). Specifically, there are differences in genetic counselors' and interpreters' perceived responsibilities during sessions relating to advocacy, psychosocial, and cultural domains (Wang et al., 2022). Interpreters may use empathic linguistic tools like contextualization, encouragement, checking comprehension, endearment, and softening as a means of culturally tailoring the information spoken by the healthcare provider before delivering it to the patient (Gutierrez et al., 2019). While this may be beneficial from the patient perspective, non‐verbatim interpretation may contribute to healthcare providers having limited trust in professional interpreters, specifically in terms of the accuracy of information they convey to patients (Jaeger et al., 2019; Lara‐Otero et al., 2018). Lack of trust in the interpreter was reported by genetic counselors as one of the main constraints on their relationship with interpreters (Wang et al., 2022). Interestingly, our study showed that genetic counselors' perceived dialogue engagement with the patient increased with higher perceived levels of trust in the interpreter. Perhaps genetic counselors who have less trust in interpreters and in the accuracy of their interpretation feel the need to repeat and re‐explain information during a session, thus hindering the openness of dialogue and quality of communication, and thus also contracting quality, with the patient.

Surprisingly, our results also indicated that there was lower perceived ability to understand patient expectations and perceptions for genetic counselors who believe interpreter judgment should be respected during the session than in those who do not believe or are unsure if interpreter judgment should be respected. Previous research indicates that interpreters often culturally and psychosocially tailor the information they deliver to patients through a variety of linguistic tools (Gutierrez et al., 2019; Wang et al., 2022). Thus, one would expect that when genetic counselors respect interpreters' judgment and embrace their decisions to utilize these tools, their ability to elicit and understand patient expectations would be enhanced, which was the opposite of what our results showed. One potential explanation could be that genetic counselors who do not respect interpreters' judgment overestimate their own ability to achieve this component of contracting, as they feel they do not need much assistance from interpreters. Unfortunately, since our data were confined to genetic counselors' perceptions, there is not enough information to adequately interpret this result. Overall, it is not clear how exactly the genetic counselor‐interpreter relationship impacts dialogue engagement and contracting, but our results suggest there is an association that warrants further evaluation.

### 4.3 Limitations

The outcome measure for contracting success that was designed and utilized in this study was novel, and thus unvalidated, which raises the question of whether it truly assessed contracting success. Our results indicated that genetic counselors' perceived contracting success, dialogue engagement, and sufficiency of time for the session were lower in LD than in LC sessions. These differences are consistent with the literature and indicate that the measures used in this study are in fact sensitive to LD, which adds confidence to their validity (Al Shamsi et al., 2020; Ault et al., 2019; de Leon et al., 2022; Dunlap et al., 2015; Jaeger et al., 2019; Jaramillo et al., 2016; Kamara et al., 2018; White et al., 2018; Yeheskel & Rawal, 2019). However, further replication studies are needed to validate our survey instrumentation.

Additionally, all data from this study were based solely on genetic counselors' perceptions. While surveying genetic counselors was necessary given that the time and resources available for this study hampered our ability to gather patient and interpreter perspectives, it represents a limitation. It is possible that the genetic counselors who participated in this study over‐ or underestimated their success with contracting. Additionally, how a genetic counselor perceives contracting success in a session may not coincide with how interpreters or patients themselves perceive success.

Sampling bias is another potential limitation, given that there were only 45 participants, who may have had a particular interest in this topic, thus prompting them to complete the survey. It is also possible that there was an overrepresentation of genetic counselors who believe that they are particularly successful or unsuccessful contracting with patients in LD sessions.

The sample size may have been impacted by the requirement that potential participants had completed at least one language‐discordant session in the past 12 months. As the recruitment period overlapped with the height of the COVID pandemic, this requirement may have limited the eligibility of potential participants. In addition, we only recruited from the membership of the National Society of Genetic Counselors and the Michigan Association of Genetic Counselors. Sample size might have been increased if additional recruitment had occurred via other state‐based genetic counseling organizations. Finally, the demographic composition of potential participants' patient populations could have also impacted the sample size.

Another potential limitation is recall bias. Inclusion criteria for survey participation stipulated that the most recent LD and concordant sessions must have occurred within 12 months of survey completion. This date range was chosen to maximize both participant eligibility and recall, given that some genetic counselors may not frequently work with patients with limited English proficiency. However, genetic counselors, like many healthcare providers, often see high volumes of patients, rendering their memory susceptible to imperfections with recall. Based on our data, there were also discrepancies in how recently the most recent LC session occurred compared to the most recent LD one. For example, 95.6% (43/45) of LC sessions occurred less than 1 week prior to survey uptake, while only 46.7% (21/45) of LD sessions occurred that recently (Figure S2). Thus, recall bias may have impacted genetic counselors' recollection of LD sessions more than that of LC sessions. Lastly, various interpreter modalities were utilized in the language‐discordant sessions (in‐person, telephone, video). This may have been a confounding variable, given that the literature suggests differences in communication and other patient outcomes when different types of interpretation services are used (Heath et al., 2023).

### 4.4 Generalizability

Importantly, there was no significant difference between the gender (*p* = 0.169) or race/ethnicity (*p* = 0.33) of participants in our study and that of the 2021 composition of the National Society of Genetic Counselors (NSGC) (Figure S5). This suggests that these results may be generalizable to the broader genetic counselor population, even though the sample size was small (*n* = 45).

The National Society of Genetic Counselors (NSGC) Code of Ethics explicitly states that genetic counselors should “provide genetic counseling services to their clients regardless of their clients'… language.” The framework of genetic counseling practice emphasizes that these services should be tailored and patient‐centered (Doyle et al., 2016; Redlinger‐Grosse et al., 2017; Veach et al., 2007). However, the results of our study suggest that LD patients may be receiving suboptimal genetic counseling care in terms of quality of communication and personalization via contracting. Our findings clearly highlight the importance of genetic counselors actively engaging in reciprocal conversation and interchange with patients who do not communicate using the same language that they do. If possible, genetic counselors should also consider allotting more time for LD sessions and meet with the interpreter prior to the session, which is the first step of establishing a trusting and mutually respectful relationship with them. This meeting also provides an opportunity to review the case and clarify questions that the interpreter has, with the hope of making the communication more seamless with the patient during the session. Wang et al. (2022) found that one of the top constraints reported by interpreters that impacts their relationship with genetic counselors is insufficient information pre‐session from the genetic counselor. Additionally, 90% of interpreters in that study stated that they would want to receive resources from a genetic counselor (Wang et al., 2022). A pre‐session meeting would provide a wonderful opportunity for both rapport building and information sharing between the genetic counselor and interpreter. In fact, the International Medical Interpreters Association (IMIA, n.d.) Guide on Working with Medical Interpreters recommends that healthcare providers meet with the interpreter before the session for these exact reasons.

Technical terminology is another factor cited by interpreters that makes interpretation of clinical genetics content difficult (Krieger et al., 2018). In fact, genetic counselors have reported that interpreters often need time to clarify complex genetics concepts with them during the session before delivering messages to patients, which contributes to time constraints (Wang et al., 2022). Thus, pursuing opportunities to educate interpreters on complicated genetics topics and on the role of genetic counselors may improve interpreters' confidence during sessions, which could ultimately minimize time constraints and maximize communication efficiency during genetic counseling sessions. Seminars and webinars have been cited by interpreters as the most desired resource from genetic counselors (Wang et al., 2022). Developing formal, standardized guidelines on how to train genetic counseling students to work with interpreters could also be beneficial, given that no such guidelines currently exist (Accreditation Counsil for Genetic Counseling, 2019). Lastly, efforts to increase the linguistic diversity of genetic counselors in the field will limit LD care and likely improve patient satisfaction, given that research suggests patients feel relieved and thankful to have a genetic counselor who communicates in their language (de Leon et al., 2022). A recent study showed that even genetic counselors who are not fluent, but have either basic or fair proficiency in another language can be helpful in improving communication, which may reduce health disparities for clients who communicate in that language (Waggoner et al., 2023).

Given that our data were limited to genetic counselors' perspectives, an important next step would be to gain perspectives on this issue from patients and interpreters. In particular, there would be a benefit to further elucidating how exactly the relationship between the genetic counselor and interpreter plays a role in communication and contracting between the genetic counselor and patient, likely in a qualitative context, with interviews or focus groups. It is also possible that the type of service delivery and type of interpreter (in‐person vs. video vs. telephone) could impact genetic counselors' contracting success with the patient and relationship with the interpreter, which should be further investigated in future studies. Additionally, this study focused on contracting, but there are likely many other aspects of a genetic counseling session that are impacted by LD that could result in disparities in the quality of care being delivered to patients with LEP that could be studied.

Lastly, it is impossible to fully disentangle language from culture. While our study did not specifically address how cultural factors influence communication and contracting in LD sessions, previous studies suggest culture is critical in this space (Agather et al., 2017; Krieger et al., 2018; Lor et al., 2016; Luksic et al., 2020; Soled, 2020). In fact, many Spanish‐speaking genetic counselors who took part in a qualitative study believed that genetic counseling sessions are influenced more by factors like culture and health literacy than by language concordance (de Leon et al., 2022). Also, medical interpreters have identified significant “culture bumps” and gaps in cultural competency that can negatively impact rapport building between genetic counselors and their patients with LEP (Rosenbaum et al., 2020). The results of our study emphasized the importance of actively communicating with patients with LEP to facilitate tailored counseling and patient‐centered care. However, there is evidence suggesting that communication preferences also vary by culture (Hawley & Morris, 2017; Krieger et al., 2018). This further emphasizes the importance of continued research on how genetic counselors should consider culture to most appropriately communicate and contract with their patients.

## AUTHOR CONTRIBUTIONS

Anna Burton has made substantial contributions to the conception/design of the work, the acquisition, analysis, and interpretation of data for the work, the drafting of the work, and the final approval of the version to be published. She agrees to be accountable for all aspects of the work in ensuring that questions related to the accuracy or integrity of any part of the work are appropriately investigated and resolved. Dana Schlegel has made substantial contributions to the conception/design of the work, the acquisition, analysis, and interpretation of data for the work, the revision of the work, and the final approval of the version to be published. She agrees to be accountable for all aspects of the work in ensuring that questions related to the accuracy or integrity of any part of the work are appropriately investigated and resolved. Charité Ricker has made substantial contributions to the conception/design of the work, the acquisition, analysis, and interpretation of data for the work, the revision of the work, and the final approval of the version to be published. She agrees to be accountable for all aspects of the work in ensuring that questions related to the accuracy or integrity of any part of the work are appropriately investigated and resolved. Beverly M. Yashar has made substantial contributions to the conception/design of the work, the acquisition, analysis, and interpretation of data for the work, the revision of the work, and the final approval of the version to be published. She agrees to be accountable for all aspects of the work in ensuring that questions related to the accuracy or integrity of any part of the work are appropriately investigated and resolved.

## FUNDING INFORMATION

Rackham Graduate Student Research Award.

## CONFLICT OF INTEREST STATEMENT

Author Anna Burton, Dana Schlegel, Charité Ricker, and Beverly M. Yashar declare that they have no conflict of interest.

## Supporting information


Data S1.

## Data Availability

The data that support the findings of this study are available from the author upon request.
