# Supplementary material for: The impact of language discordance on genetic counselors' ability to establish a working alliance with patients
Source: J Genet Couns. 2025 Apr 30;34(3):e70019. doi: 10.1002/jgc4.70019 (PMC12043036; doi:10.1002/jgc4.70019)
Supplement: Supplementary file 1 — Data S1. [file JGC4-34-0-s001.docx]

**SUPPLEMENTAL MATERIALS**

**
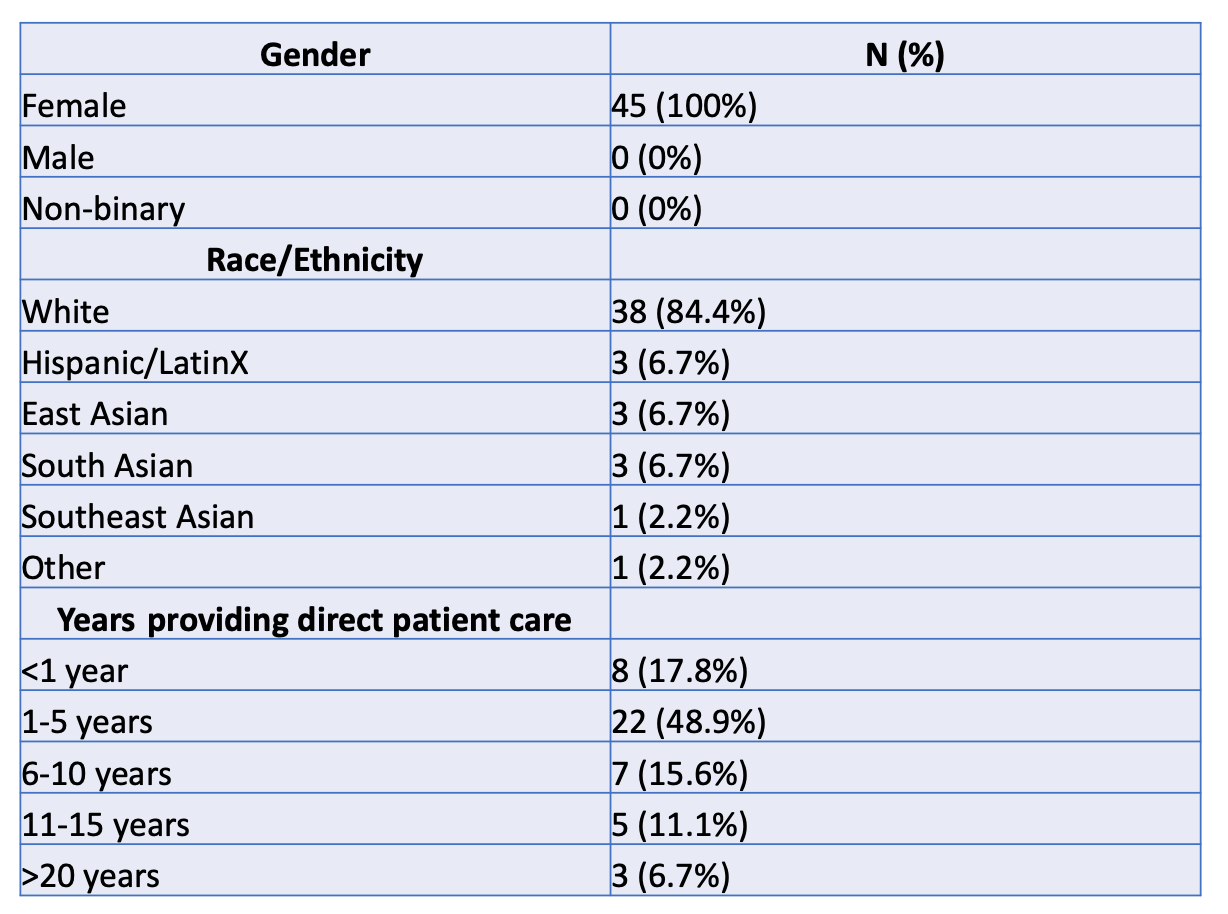
**

**Figure S1. Participant demographics and experience providing patient care.**

**
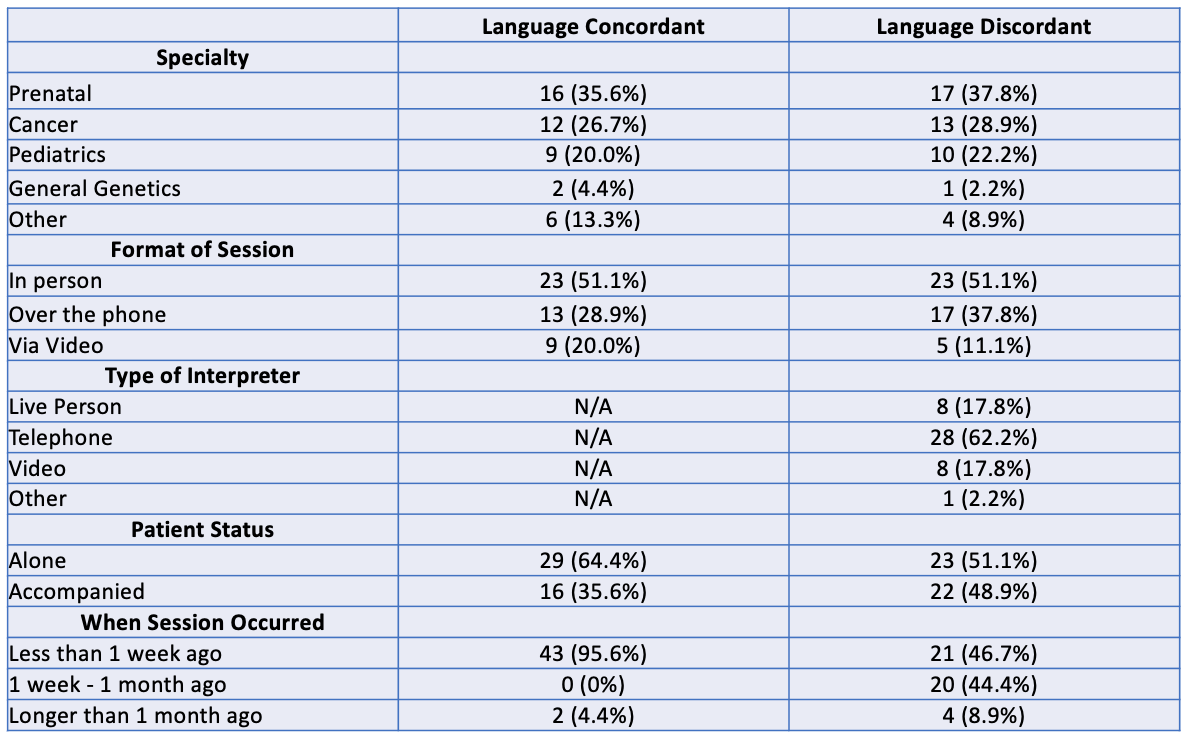
**

**Figure S2. Characteristics of language-discordant and language-concordant sessions.**

**
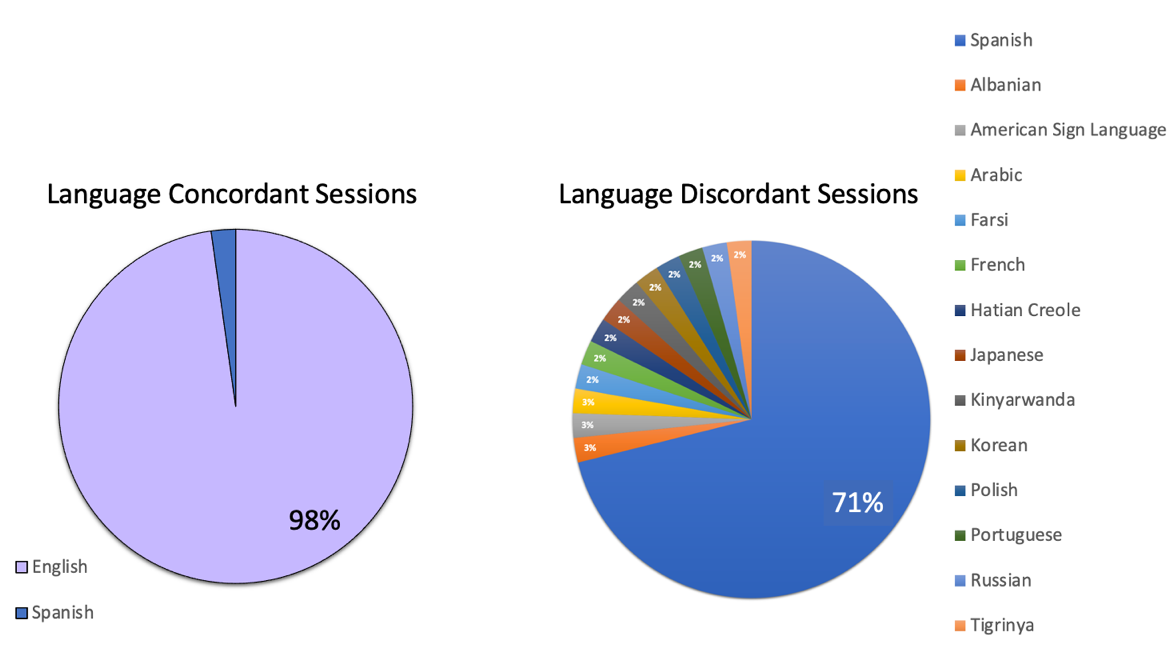
**

**Figure S3. Languages in which LC and discordant sessions were conducted.**

**
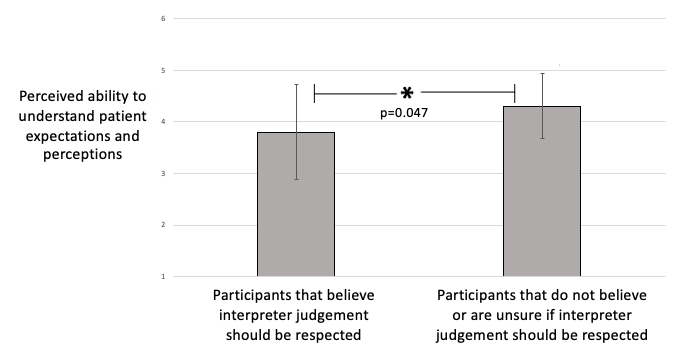
**

**Figure S4. Beliefs on respecting interpreters’ judgement is associated with success in one component of contracting.** Genetic counselors’ who answered “false” or “unsure” to the statement “During a session, if the interpreter insists that a question is inappropriate, his/her judgement should be respected” had a significantly higher perceived ability to understand the primary communicators’ expectations and perceptions than did the genetic counselors who answered “true” (p=0.047).

| **Demographic** | **NSGC PSS 2021 (n=2,993)** | **Study Population (n=45)** |
| --- | --- | --- |
| **Gender** |  |  |
| Female | 2828 (~94%) | 45 (100%) |
| Male | 151 (~5%) | 0 (0%) |
| Non-binary/ third gender, preferred not to respond, preferred to self-describe | 14 (<1%) | 0 (0%) |
|  |  |  |
| **Race/Ethnicity/Culture** |  |  |
| Non-Hispanic White | 2619 (87.5%) | 36 (80%) |
| All others | 374 (12.5%) | 9 (20%) |

**Figure S5. Study participant and National Society of Genetic Counselor (NSGC) demographics.** There was no significant difference between the gender (p=0.169) or race/ethnicity (p=0.33) of participants in our study and that of the 2021 composition of the National Society of Genetic Counselors (NSGC).


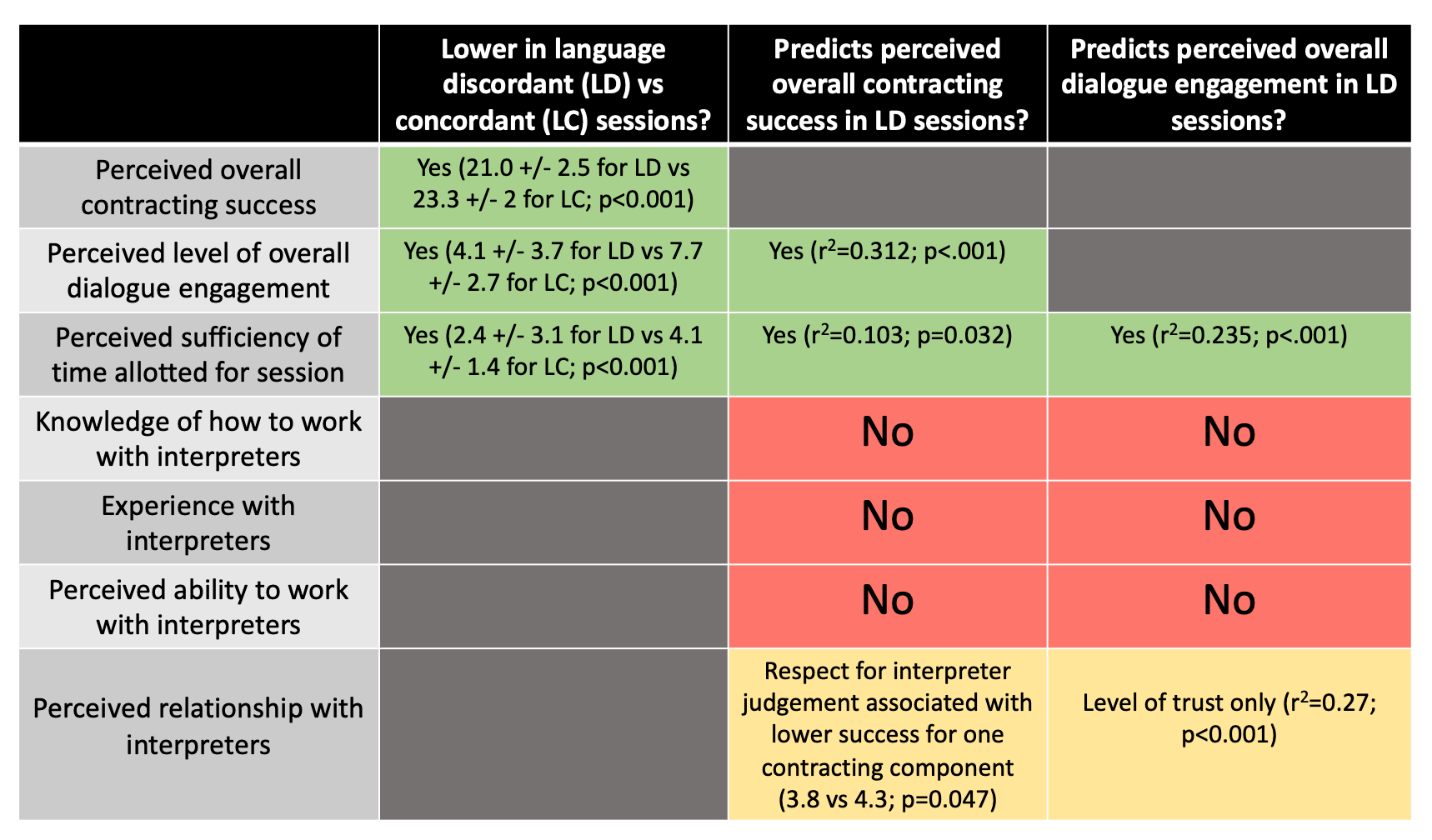


**Figure S6. Summary of Results.**

**Survey Instrument**

This study has been approved by the IRB (HUM00206655). The purpose of this study is to explore various factors that may impact genetic counselors’ ability to establish a mutually-agreed upon agenda with patients when professional medical interpreters are used. To participate in this study:

1. You must be a board-certified genetic counselor who provides direct patient care or provided direct patient care in the past.
2. You must have used a professional medical interpreter* (either in person, over the phone, or via video) to counsel a patient within the past 12 months.

*For the purposes of this study, professional medical interpreters are defined as individuals who are trained in medical interpretation and appointed or contracted by a hospital, medical center, or health care clinic to assist in communicating with patients who have limited proficiency in the language spoken by the provider, or who are Deaf or hard of hearing. Therefore, sessions involving the use of a professional medical interpreter of American Sign Language (ASL) or any spoken language can be included. Family members or friends of a patient who assist with interpretation during a session are not considered professional medical interpreters.

Participation in this study is voluntary, and the information you share will be kept confidential. Participating in this study may not benefit you directly, but it may help us learn about ways through which interpreter-mediated genetic counseling services can be improved in the future. If you participate in this study, you will be entered into a raffle to win one of twenty-five $20 gift cards. By completing this survey, you are consenting to participate in this study.

Following survey completion, you will be asked if you are willing to participate in a semi-structured interview to gather additional information about your perspectives on factors that impact your ability to establish mutually agreed-upon agendas with patients when interpreters are used. If you participate in an interview, you will be given an additional $40 gift card.

If you have any questions about this study, please contact Anna Burton at [akburton@umich.edu](mailto:akburton@umich.edu).

**Eligibility questions**

1. Are you a board-certified genetic counselor who provides direct patient care or provided direct patient care in the past?
2. Yes
3. No
4. Have you used a professional medical interpreter to counsel a patient within the past 12 months?
   1. Yes
   2. No

If no to 1 or 2: “You unfortunately are not eligible to participate in this study. Thank you for your interest and time.”

**Please answer the following questions about yourself:**

1. Please select your gender from the list below:
2. Man
3. Woman
4. Trans man
5. Trans woman
6. Non-binary
7. Other (free text)
8. I prefer not to answer
9. Please select your race/ ethnicity/ culture from the list below (choose all that apply)
10. White
11. Hispanic/Latinx
12. East Asian
13. South Asian
14. Black/African American
15. West Asian/Middle Eastern/North African
16. Southeast Asian
17. Native American/Alaska Native, First Nations
18. Native Hawaiian/Pacific Islander
19. Other (free text)
20. I prefer not to answer
21. Including English, how many languages do you speak (or sign) fluently?
22. 1
23. 2
24. 3 or more
25. (only if question 3 is more than 1) Are you a native English speaker?
    1. Yes
    2. No
26. (only if question 3 is more than 1) Which languages other than English do you speak (or sign) fluently? (free text)
27. For how many years have you provided direct patient care as a genetic counselor?
28. less than 1 year
29. 1-5 years
30. 6-10 years
31. 11-15 years
32. 16-20 years
33. >20 years
34. Approximately how many times have you worked with a professional medical interpreter (including phone, video, or in-person) over the past month?
35. 0-5
36. 6-10
37. 11-15
38. >15
39. Approximately how many times have you worked with a professional medical interpreter (including phone, video, or in-person) over the past year?
40. 1-10
41. 11-50
42. 51-100
43. >100

**Please answer the following questions about how providers should work with professional medical interpreters.**

1. When working with an interpreter, you should address your client in third person (i.e., “please ask him what medications he is taking”) rather than in first person (i.e., “what medications are you taking?”)
2. True
3. False
4. I am not sure
5. When working with an interpreter, it is important to use simple language and avoid jargon, technical terms, and slang so the interpreter will be able to interpret more easily.
6. True
7. False
8. I am not sure
9. When working with an in-person, spoken-language interpreter, you should allow the interpreter to best position themselves to promote direct eye contact between ----- wherever possible
10. Provider and patient
11. Provider and interpreter
12. Patient and interpreter
13. I am not sure
14. During a session, if the interpreter insists that a question is inappropriate, his/ her judgement should be respected
15. True
16. False
17. I am not sure

**Please reflect on the last time you counseled a patient using a professional medical interpreter* (either in person, over the phone, or via video). This will be referred to as your “most recent language-discordant session.” Answer the following questions with that particular session in mind.**

***Professional medical interpreters are defined as individuals who are trained in medical interpretation and appointed or contracted by a hospital, medical center, or health care clinic to assist in communicating with patients who have limited proficiency in the language spoken by the provider, or who are Deaf or hard of hearing. Therefore, sessions involving the use of a professional medical interpreter of American Sign Language (ASL) or any spoken language can be included. Family members or friends of a patient who assist with interpretation during a session are not considered professional medical interpreters.**

1. When was your most recent language-discordant session?
2. Less than 1 week ago
3. 1 week-1 month ago
4. Longer than one month ago
5. In which type of clinic did you see this patient?
6. Prenatal
7. Cancer
8. Pediatrics
9. General Genetics
10. Other (free text)
11. Did the patient attend the session alone?
12. Yes
13. No, they were accompanied by a friend or family member
14. What form of an interpreter did you use during this session?
15. Live person
16. Telephone
17. Video
18. Other (i.e. Communication access real-time translation or CART)
19. Had you previously worked with the professional medical interpreter that was used in your most recent language-discordant session?
20. yes, once
21. yes, a few times
22. yes, many times
23. no, never
24. not sure/ can’t remember
25. Please move the sliding bar anywhere along the scale to indicate how you felt when working with the professional medical interpreter you used in your most recent language-discordant session.
26. well-trained/ not well-trained
27. confident/ unsure
28. Please move the sliding bar anywhere along the scale to indicate how you would describe the relationship you had with the professional medical interpreter used during your most recent language-discordant session
29. collaborative/ noncollaborative
30. trusting/ distrusting

**Please answer the following questions about the primary communicator in your most recent language-discordant session. The primary communicator is the person with whom you interacted the most throughout the session. This person may be the patient or a family member or friend of the patient.**

1. During this session, were you and the primary communicator interacting in person, over the phone, or via video?
2. In person
3. Over the phone
4. Via video
5. What language was used by the primary communicator during this session? (free text)
6. Please move the sliding bar anywhere along the scale to indicate how you would describe the dialogue between yourself and the primary communicator during your most recent language-discordant session
7. Interactive/ one-sided
8. Conversational/ rigid
9. Please select the primary communicator’s gender from the list below:
10. Man
11. Woman
12. Trans man
13. Trans woman
14. Non-binary
15. Other (free text)
16. My patient preferred not to answer so I do not know
17. I do not remember
18. Please select the primary communicator’s race/ ethnicity/ culture from the list below (choose all that apply)
19. White
20. Hispanic/Latinx
21. East Asian
22. South Asian
23. Black/African American
24. West Asian/Middle Eastern/North African
25. Southeast Asian
26. Native American/Alaska Native, First Nations
27. Native Hawaiian/Pacific Islander
28. Other (free text)
29. I do not remember
30. Please select the primary communicator’s approximate age.
31. Younger than 20
32. 20-29
33. 30-39
34. 40-49
35. 50-59
36. 60-69
37. 70 or older
38. I do not remember
39. For your most recent language-discordant session, rate your level of agreement with each statement by selecting one of the 5 options (strongly disagree, somewhat disagree, neither agree nor disagree, somewhat agree, or strongly agree).
40. I was able to understand the primary communicator’s expectations and perceptions
41. I was able to discern the primary communicator’s knowledge about the referring indication
42. I was able to understand the primary communicator’s concerns
43. The agenda that was developed was mutually agreed-upon based on the primary communicator’s expectations, perceptions, knowledge, and concerns
44. The agenda was modified based on emerging concerns of myself and the primary communicator throughout the session
45. Please move the sliding bar anywhere along the scale to indicate how you would describe the amount of time allotted for your most recent language-discordant session.
46. Sufficient/ insufficient
47. Please comment on any factors you feel impacted your ability to build rapport and contract with the primary communicator during your most recent language-discordant session. (free text)

**Please reflect on the last time you counseled a patient who communicated in the same language as you. This session should NOT have required a professional medical interpreter nor any other person or device to assist with interpretation/ translation during the visit.  This session will be referred to as your “most recent language-concordant session.” Answer the following questions with that particular session in mind.**

1. When was your most recent language-concordant session?
2. Less than 1 week ago
3. 1 week- 1 month ago
4. Longer than 1 month ago
5. In which type of clinic did you see this patient?
6. Prenatal
7. Cancer
8. Pediatrics
9. General Genetics
10. Other (free text)
11. Did the patient attend the session alone?
12. Yes
13. No, they were accompanied by a friend or family member

**Please answer the following questions about the primary communicator in your most recent language-concordant session. The primary communicator is the person with whom you interacted the most throughout the session. This person may be the patient or a family member or friend of the patient.**

1. During this session, were you and the primary communicator interacting in person, over the phone, or via video?
2. In person
3. Over the phone
4. Via video
5. What language was used by you and the primary communicator during the session? (free text)
6. Please move the sliding bar anywhere along the scale to indicate how you would describe the dialogue between yourself and the primary communicator during your most recent language-concordant session
7. Interactive/ one-sided
8. Conversational/ rigid
9. Please select the primary communicator’s gender from the list below:
10. Man
11. Woman
12. Trans man
13. Trans woman
14. Non-binary
15. Other (free text)
16. My patient preferred not to answer so I do not know
17. I do not remember
18. Please select the primary communicator’s race/ ethnicity/ culture from the list below (choose all that apply)
19. White
20. Hispanic/ Latinx
21. East Asian
22. South Asian
23. Black/African American
24. West Asian/Middle Eastern/North African
25. Southeast Asian
26. Native American/Alaska Native, First Nations
27. Native Hawaiian/Pacific Islander
28. Other (free text)
29. I do not remember
30. Please select the primary communicator’s approximate age
31. <20
32. 20-29
33. 30-39
34. 40-49
35. 50-59
36. 60-69
37. >70
38. I do not remember
39. For your most recent language-concordant session, rate your level of agreement with each statement by selecting one of the 5 options (strongly disagree, somewhat disagree, neither agree nor disagree, somewhat agree, or strongly agree).
40. I was able to understand the primary communicator’s expectations and perceptions
41. I was able to discern the primary communicator’s knowledge about the referring indication
42. I was able to understand the primary communicator’s concerns
43. The agenda that was developed was mutually agreed-upon based on the primary communicator’s expectations, perceptions, knowledge, and concerns
44. The agenda was modified based on emerging concerns of myself and the primary communicator throughout the session
45. Please move the sliding bar anywhere along the scale to indicate how you would describe the amount of time allotted for your most recent language-concordant session
46. Sufficient/ insufficient
47. Please comment on any factors you feel impacted your ability to build rapport and contract with the primary communicator during your most recent language-concordant session. (free text)

**Post-survey questions**

1. Would you like to be entered into a raffle to win one of twenty-five $20 monetary rewards?
2. Yes. My email is:
3. No.
4. Are you willing to be contacted in order to participate in a semi-structured interview? The interview will serve to gather additional perspectives you have about the factors that impact your ability to establish mutually-agreed upon agendas with patients when interpreters are used. Please note that interview participants will be given an additional $40 monetary reward.
5. Yes. My email is:
6. No
